# The cAMP-signaling cancers: Clinically-divergent disorders with a common central pathway

**DOI:** 10.3389/fendo.2022.1024423

**Published:** 2022-10-13

**Authors:** Graeme B. Bolger

**Affiliations:** BZI Pharma LLC, Birmingham, AL, United States

**Keywords:** cAMP, PKA, phosphodiesterase, adenylyl cyclase, CREB, Cushing, Carney Complex

## Abstract

The cAMP-signaling cancers, which are defined by functionally-significant somatic mutations in one or more elements of the cAMP signaling pathway, have an unexpectedly wide range of cell origins, clinical manifestations, and potential therapeutic options. Mutations in at least 9 cAMP signaling pathway genes (*TSHR, GPR101, GNAS, PDE8B, PDE11A, PRKARA1, PRKACA, PRKACB*, and *CREB*) have been identified as driver mutations in human cancer. Although all cAMP-signaling pathway cancers are driven by mutation(s) that impinge on a single signaling pathway, the ultimate tumor phenotype reflects interactions between five critical variables: (1) the precise gene(s) that undergo mutation in each specific tumor type; (2) the effects of specific allele(s) in any given gene; (3) mutations in modifier genes (mutational “context”); (4) the tissue-specific expression of various cAMP signaling pathway elements in the tumor stem cell; and (5) and the precise biochemical regulation of the pathway components in tumor cells. These varying oncogenic mechanisms reveal novel and important targets for drug discovery. There is considerable diversity in the “druggability” of cAMP-signaling components, with some elements (GPCRs, cAMP-specific phosphodiesterases and kinases) appearing to be prime drug candidates, while other elements (transcription factors, protein-protein interactions) are currently refractory to robust drug-development efforts. Further refinement of the precise driver mutations in individual tumors will be essential for directing priorities in drug discovery efforts that target these mutations.

## cAMP signaling: A regulator of cell growth and proliferation in disparate cells and tissues

The cAMP-signaling cancers are a set of clonal proliferative neoplasms characterized by the presence of driver mutations in components of the cAMP pathway. Recent genetic, molecular, cellular and clinical findings have provided novel and actionable insights into how a very diverse set of human cancers can arise from mutations in a single regulatory pathway. These insights in turn have reinforced the importance of understanding fully the structure and regulation of each of the components of the cAMP-signaling pathway and, equally importantly, provided a new impetus for drug discovery efforts targeting key elements of the pathway. It has become increasingly likely that drugs targeting cAMP-signaling components will be entering clinical trials in a broad range of human cancers and, potentially, into routine clinical use in oncology. This review will focus on recent developments in the functional characterization of cAMP-signaling pathway mutations in human cancer and on rational drug-discovery programs focused on these mutations.

## Essential components of cAMP signaling pathways in cancer

**Figure 1:**
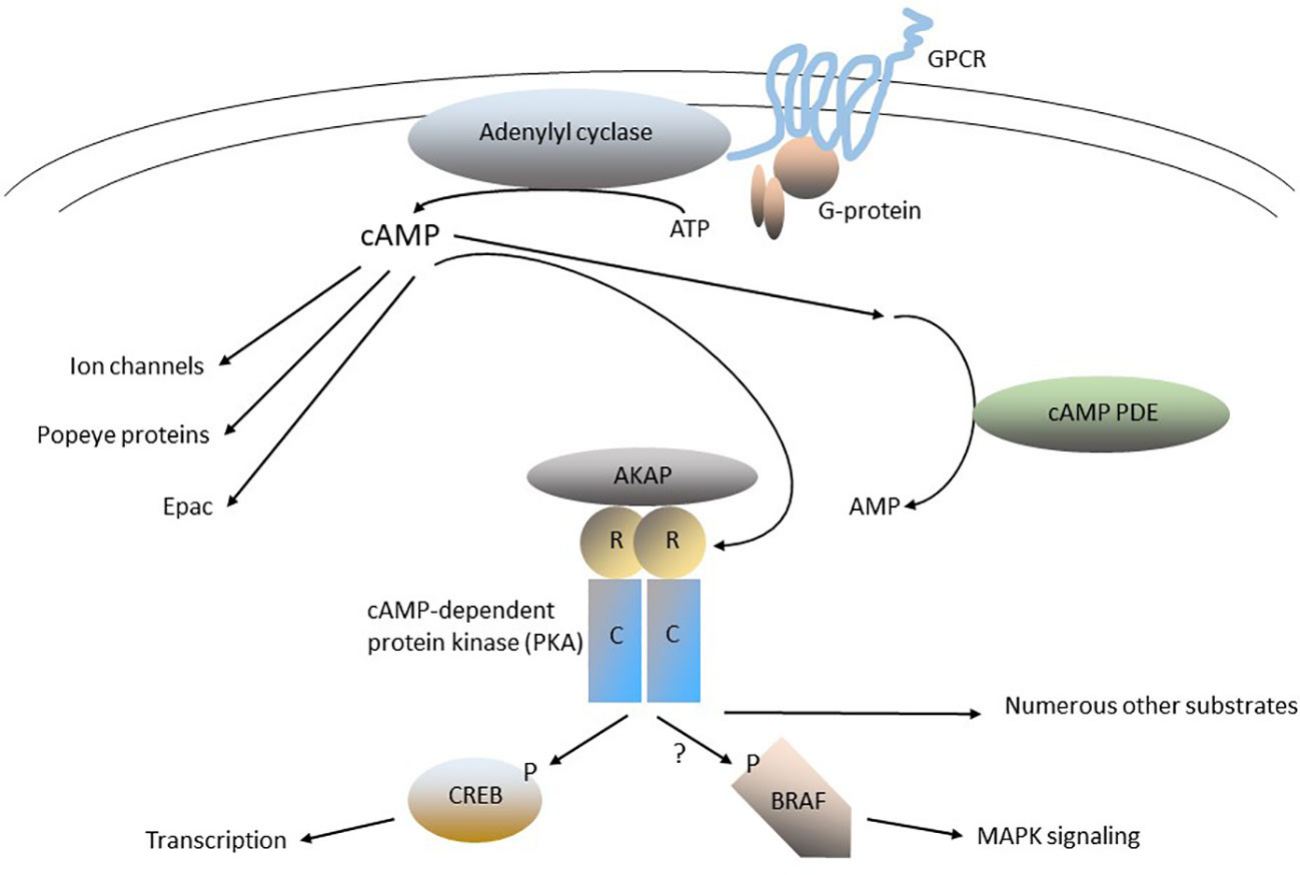
The cAMP-signaling pathway. G-protein-coupled receptors (GPCRs) are a large family of 7-helix transmembrane proteins that are the physiological receptors for circulating hormones (e.g., TSH, ACTH, FSH, and LH, among many others) and neurotransmitters that bind to their extracellular regions (the plasma membrane is indicated by the pair of curved lines). Orphan GPCRs (e.g., that encoded by *GPR101*), encode GPCRs for which the physiologic ligand has yet to be determined. GPCRs interact with trimeric G-proteins by recruiting them to specific regions on their intracellular loops. G-proteins have three subunits (α, β, or γ) and the members of the α family can be divided into stimulatory (Gαs) or inhibitory (Gαi) isoforms (e.g., *GNAS* is one of several genes encoding Gαs isoforms). Gα and/or Gβγ bind to, and regulate, one of several downstream effectors, including membrane-associated adenylyl cyclase, which catalyzes the synthesis of cAMP from ATP. cAMP is a soluble “second messenger” that can diffuse widely in cells. cAMP phosphodiesterases (PDEs) catalyze the hydrolysis (breakdown) of cAMP and thereby play a central in regulating cAMP signaling in cells. cAMP can activate several targets, including the cAMP-dependent protein kinase (protein kinase A, PKA), EPAC, Popeye proteins and ion channels. The PKA holoenzyme is a tetramer of 2 catalytic subunits (C-subunits, Cα or Cβ), and 2 regulatory subunits (R-subunits, RIα, RIβ RIIα or RIIβ). The subcellular localization of PKA is determined by its binding to A-kinase anchoring proteins (AKAPs). In the inactive PKA holoenzyme, the R-subunits bind tightly to the C-subunits and inhibit their activity. cAMP activates PKA by binding to the R-domains, producing a conformational change that activates the C-domains. PKA is a serine-threonine protein kinase that can phosphorylate numerous substrates, depending on the specific cell type and subcellular location. The cAMP-responsive element binding protein (CREB) is an important substrate and downstream target of PKA; it regulates the transcription of numerous genes. The oncoprotein BRAF is regulated in part by PKA, as well as other signaling proteins, such as RAS proteins and tyrosine-protein kinases.

The cyclic nucleotide, 3’, 5’ cyclic adenosine monophosphate (cAMP) is a prototypical small molecule intracellular “second messenger” (**Figure 1**). In the classical paradigm of second-messenger signaling, cAMP is synthesized in response to extracellular stimuli by membrane-associated adenylyl cyclase, and then diffuses throughout the cell, where it interacts with specific downstream effector proteins (1–3). Among the most important regulators of membrane-associated adenylyl cyclase are numerous G-protein-coupled receptors (GPCRs), which regulate adenylyl cyclase through trimeric GTP-binding proteins (G-proteins). There are numerous variations on the classical paradigm: for example, cAMP can also be generated by soluble forms of adenylyl cyclase, located in diverse cellular sub-fractions (4, 5). Another essential set of cAMP pathway regulatory enzymes are the cyclic nucleotide phosphodiesterases (PDEs), which hydrolyze (degrade) cAMP (and/or cGMP) and thereby modulate its levels in cells (1, 2, 6–9). Physiologically-important cAMP effectors include cAMP-specific protein kinase (protein kinase A, PKA), cyclic nucleotide-gated ion channels, exchange proteins activated by cAMP (EPACs), and Popeye proteins (10–19). The various pathway components also mediate “cross-talk” between cAMP signaling and other cellular pathways, particularly the MAPK pathway. Both synthesis and breakdown of cAMP can be highly localized in cells, producing “compartments”, “pools” or “gradients” where its concentration is tightly regulated in space and time (20–23). The overall process is highly dynamic, with short- and long-term feedback loops that adjust the “gain” of various components of the pathways and increase their versatility and range of response.

## cAMP signaling in cancer: The importance of driver mutations

Key to determining the physiological, pathologic and clinical importance of cAMP signaling in cancer is the concept of driver mutations. Driver mutations in cancer are defined as germline or somatic mutations (changes in DNA sequence) in tumor cells that play an essential role in tumorigenesis (24–28). Typically, driver mutations have the following characteristics: (1) they affect the expression or structure of the protein and/or RNA encoded by the mutated gene(s) and thereby produce a change in the physiological function(s) of the gene product, leading to a growth advantage; (2) they are localized in specific regions of the gene product (“hot spots”) essential to its biochemical function, such as its enzymatic activity, its regulation, or its ability to regulate other cell components; and (3) the specific mutation can be detected in a high proportion of clinical specimens obtained from any specific cancer type. In contrast, “passenger” mutations in tumor cells have an uncertain role in tumorigenesis; they typically do not change the physiological or biochemical functions of the gene product, do not concentrate in “hot spots”, and are found in only a small proportion of clinical specimens obtained from any specific cancer type. Each of the cAMP-signaling cancers discussed in this review contains driver mutations that meet these criteria, as described in detail below. Throughout this review, when a capitalized term is italicized, it refers to the gene of interest, while capitals without italics refer to the protein product encoded by that gene.

## Wide variation in the biology and clinical features of cAMP-signaling cancers

To date, at least 9 different genes encoding components of the cAMP-signaling pathway have been shown to develop driver mutations in human cancer: (a) Mutations in the thyroid-stimulating-hormone receptor (TSHR; a GPCR) in thyroid adenomas; (b) Mutations in GPR101, an orphan GPCR, in pituitary tumors; (c) Mutations in GNAS, a trimeric G-protein, in a range of endocrinopathies and endocrine cancers; (d) Mutations in two different phosphodiesterases, PDE11A and PDE8B, in adrenal hyperplasia/adenomas and endocrine tumors of the testis; (e) Mutations of the PKA regulatory and two different catalytic subunits in adrenal adenomas and other disorders; (f) Mutations of the PKA catalytic subunit in fibrolamellar hepatocellular carcinoma (HCC); (g) mutations in the cAMP-response element binding protein (CREB) in several rare cancers (**Table 1**). The precise role of each of these genes in their respective disorder(s) is described in detail in the subsequent sections. The pathway and some of these cancers have also been the subject of several recent reviews (29, 30).

**Table 1:** Genes encoding cAMP signaling pathway components that develop driver mutations in human cancer. All abbreviations are defined in the text.

Gene	Pathway component	Tissue/organ involved	Tumor type
*TSHR*	GPCR	Thyroid	Adenomas
*GPR101*	GPCR	Pituitary	Somatotropinomas
*GNAS*	G protein alpha subunit	Adrenal cortex; Parathyroid	Adenomas
*PDE8B*	Phosphodiesterase	Adrenal cortex	Adenomas
*PDE11A*	Phosphodiesterase	Adrenal cortex; Testis Leydig/Sertoli cells; Germ cells	Adenomas; Germ cell tumors
*PRKARA1*	PKA, regulatory subunit	Adrenal cortex; Testis Sertoli cells; Pituitary	Adenomas; LCCSCT; Somatotropinomas
*PRKACA*	PKA, catalytic subunit	Adrenal cortex; Liver	Adenomas; Fibrolamellar hepatocellular cancer
*PRKACB*	PKA, catalytic subunit	Adrenal cortex	Adenomas
*CREB*	Transcription factor	Abdominal soft tissue; CNS	FET-CREB fusion tumors

### Mutations in the TSH receptor, a GPCR, in thyroid adenomas

Thyrotropin, or thyroid-stimulating hormone (TSH), is a peptide hormone produced by the anterior pituitary that has profound effects on thyroid function. The major short-term effect of TSH action is to increase production of thyroid hormone; however, over a longer term, TSH also stimulates growth and differentiation of thyroid tissue. The TSH receptor, a GPCR, regulates several downstream signaling components, including activation of adenylyl cyclase through Gαs (**Figure 1**; ref (31). An extensive variety of mutations in the TSH receptor have been identified (32, 33). Loss-of-function TSH receptor mutations impair the synthesis/release of thyroid hormone and impair the growth and differentiation of thyroid tissue (32). Gain-of-function TSH receptor mutations serve as driver mutations in thyroid adenomas, where they increase the synthesis/release of thyroid hormone and stimulate the growth and differentiation of thyroid tissue (32). The phenotypic effects of gain-of-function TSH receptor mutants is generally felt to require, at least in part, activation of PKA and the stimulation of gene transcription by CREB (31). The net effect of gain-of-function TSH mutations in thyroid adenomas, particularly when combined with other driver mutations (34–37) is strongly pro-proliferative and anti-apoptotic, leading to the development of the tumor phenotype in these cells (32). Consistent with this model, recent data have implicated the TSH receptor in other malignancies, notably ovarian carcinomas, where it serves as the receptor for thyrostimulin, a pro-proliferative peptide growth factor (31). These observations have led to the development of therapies that target the TSH receptor in thyroid cancer (and potentially other cancers), including nanoparticles that bind the receptor and target cell destruction (38).

### Mutations in GPR101, a GPCR, in pituitary tumors

GPR101 is an orphan GPCR (i.e., its physiological ligand has yet to be determined) whose expression is limited to specific regions of the adult human brain (39). It is expressed in human fetal and adolescent pituitary tissue, but is minimally expressed in adult pituitary. It appears to be coupled to Gαs and activate a cAMP reporter element (CRE) when overexpressed in cells, suggesting that it stimulates adenylyl cyclase (40). Microduplications of Xq26.3, which includes GPR101, have been implicated in X-linked acrogigantism (X-LAG), an inherited disorder characterized by pituitary growth-hormone-secreting adenomas (somatotropinomas) developing in the first few years of life (41–45). However, the Xq26.3 microduplication is complex and involvement of other, nearby, genes in X-LAG cannot be excluded on the basis of current data. GPR101 mosaicism has also been implicated in the development of acquired somatotropinomas (46). Determining the physiological ligand of GPR101, and further investigation into its downstream signaling pathways, remain key objectives of further research.

### Mutations in GNAS, a G-protein α subunit, in adrenal hyperplasia and adenomas

Adrenocorticotrophic hormone (ACTH) is essential for the normal function of the cortex of the adrenal gland. ACTH is a peptide hormone that is secreted by the anterior pituitary, circulates in the blood, and binds selectively to its specific GPCR. Receptor binding of ACTH activates a stimulatory G-protein α subunit that in turn activates adenylyl cyclase, elevates intracellular levels of cAMP, and activates PKA (**Figure 1**). In the short-term, ACTH stimulates the secretion of cortisol and other steroid hormones by the adrenal cortex; in the long-term, it stimulates growth and differentiation of adrenal cortical tissue. Given the key role of cAMP normal adrenal physiology, it is not surprising that driver mutations occurring in several different cAMP signaling components can play a critical role in the development of adrenal adenomas and hyperplasia.

Among the best-studied of cAMP driver mutations in adrenal tumors are those in *GNAS*, which encodes a stimulatory G-protein α subunit that activates adenylyl cyclase. Mosaicism with an activating *GNAS* mutation is the cause of the McCune-Albright syndrome, which is characterized by numerous endocrine abnormalities, including pseudohypoparathyroidism and/or the development of adrenal hyperplasia and/or adenomas in early in life (47–51). *GNAS* mutations reduce the intrinsic GTPase of the G-protein α subunit, leading to constitutively increased adenylyl cyclase activity, elevated cAMP levels, aberrant PKA activity, and disordered phosphorylation and regulation of CREB by PKA. The resulting dysregulation of transcription, coupled with the effects of additional driver mutations, leads to a transformed phenotype characterized by cellular proliferation and anti-apoptotic activity. A different set of germline *GNAS* mutations has been linked to obesity, hormone resistance and impaired growth (52). Somatic *GNAS* mutations have been detected in a range of human cancers (53) and in fibrous dysplasia, a disorder of bone development that does not appear to progress to cancer (54).

Recent developments in drug discovery suggest that the *GNAS* G-protein α subunit may be a druggable target. Uveal melanomas are characterized by driver mutations in a different set of G-protein α subunits, encoded by *GNA11* (55, 56) and *GNAQ* (57). A cyclic depsipeptide, FR90059, that directly interacts with GNA11, preferentially inhibits downstream ERK1/2 signaling and thereby has anti-proliferative and pro-apoptotic effects on uveal melanoma cell proliferation (58, 59). Similar effects have been produced by the small-molecule inhibitor GQ127 (60). It is possible that a conceptually-similar strategy could be used to target the GNAS G-protein α subunit.

### Mutations in genes encoding adenylyl cyclases

Adenylyl cyclases, which catalyze the synthesis of cAMP from ATP (**Figure 1**), are a diverse family of proteins encoded by multiple human genes and which are involved in myriad physiological processes (1, 3, 4). Given the central role of cAMP synthesis in cAMP signaling generally, it is not surprising that an extensive search has been undertaken to identify driver mutations in adenylyl cyclases in human cancer. To date, both targeted (i.e., gene-specific) and whole-exome sequencing approaches have yielded few candidates. One study has identified potentially deleterious nonsynonymous single nucleotide polymorphisms (nsSNPs) in *ADCY6* as a prognostic or therapeutic target, but the findings have yet to be confirmed by other groups and the role of these mutations in tumorigenesis has yet to be determined (30). Further whole-exome sequencing of diverse tumor types, followed by functional analysis of the mutations identified in these screens, are necessary before we can conclude that adenylyl cyclase mutations are truly capable of acting as driver mutations in human cancer.

### Mutations in PDE11A in adrenal hyperplasia and adenomas

Several members of the PDE family play important roles in the regulation of cAMP signaling by virtue of their ability to hydrolyze (break down) cAMP (**Figure 1**). PDE11A, which hydrolyzes both cAMP and cGMP (61, 62), is expressed at high levels in adrenal cortex and also in Leydig and Sertoli cells of the testis (63). Therefore, the physiological action of PDE11A would be to antagonize ACTH action in adrenal cortical cells; conversely, loss of PDE11 function would be expected to increase cAMP levels and augment the action of ACTH in adrenal cortex. Consistent with this model, humans with germline mutations in *PDE11A* develop adrenal hyperplasia and over-production of adrenal steroids [Cushing’s syndrome; (64–71)]. Many humans with germline *PDE11A* mutations will also develop cortisol-secreting adrenal adenomas (64–66, 69).

A genome-wide scan of DNA from adrenal cortical adenoma tumor specimens from *PDE11A* mutation carriers has shown that loss of heterozygosity occurs most commonly at 2q31-2q35, which includes the *PDE11A* gene (64). These observations show that *PDE11A* can act as a recessive oncogene (tumor suppressor gene), where mutation of one allele in the germline is then followed by mutation, loss, or inactivation of the other allele in tumors [i.e., the so-called “two-hit” hypothesis; (72)]. This model is most consistent with the *PDE11A* mutations having a loss-of-function effect. Functional experiments provide support for this hypothesis: Biochemical assays have shown that disease-associated *PDE11A* mutations reduce PDE11 enzymatic activity. These mutations would therefore elevate intracellular cAMP and/or cGMP levels, activate PKA, and increase phosphorylation of CREB (64, 65, 68). In addition to increased CREB phosphorylation, activation of PKA may lead to supra-physiological phosphorylation of other substrates, including those not normally phosphorylated by PKA. The net effect of these changes would be to generate a powerful and prolonged pro-proliferative stimulus to adrenal cortical cells. They would also work in tandem with other driver mutations that have been identified in adrenal cortical adenomas, such as those affecting WNT/beta-catenin signaling (73), DNA repair, and other pathways.

### Mutations in PDE8B in adrenal hyperplasia and adenomas

PDE8 is a cAMP-specific PDE that is encoded by two separate, highly-related, genes in humans [*PDE8A* and *PDE8B*; (74)] and which can be inhibited specifically by a unique class of inhibitors (75, 76). Several protein isoforms encoded by the *PDE8B* gene, which are produced from alternatively-spliced mRNAs, appear to be selectively expressed in cells that synthesize steroid hormones, including adrenal cortex and Leydig cells (77–79). A number of reports have identified germline *PDE8B* mutations in patients with adrenal hyperplasia, adenomas and carcinomas (69, 80–82). Based on the small number of cases that have identified to date, it appears that *PDE8B*-mutation-associated adrenal tumors are more aggressive than those associated with mutations in *PDE11A*. The *PDE8B* adenoma-associated mutations attenuate PDE8 enzymatic activity (82), providing support for an essential role of *PDE8B* in adrenal function and tumorigenesis. Disease-causing mutations in *PDE8A* have not been identified, consistent with *PDE8B* having an essential function that is distinct from either *PDE8A* or *PDE11A*.

### Potential role of mutations in other PDE genes in adrenal tumors

The routine application of whole-exome-sequencing continues to expand our knowledge of potential driver mutations in adrenal tumors. One relatively-recent study has identified germline mutations in 9 (24%) of 37 children, involving *PDE5A* (2 patients), *PDE11A* (2 patients), *PDE4DIP* (a putative PDE4-interacting protein, 2 patients), *PDE3B*, *PDE6B*, and *PDE8A* (one patient each; ref (71).). Another recent study identified germline mutations in *PDE2A* or *PDE3B* in individuals with bilateral adrenal hyperplasia and familial primary aldosteronism (83). Analysis of the functional effects of these mutations in enzymatic, cell-based and animal models should provide additional confirmation of the role(s) of these genes in adrenal tumorigenesis and potentially in other cancers.

### Mutations in PDE11A and tumors of the testis

In addition to its roles in thyroid and adrenal signaling, cAMP signaling is vital to the function of Leydig and Sertoli cells in the human testis. Testosterone and closely-related sterols are synthesized in Leydig cells, while the growth and differentiation of germ cells (i.e., the precursors of sperm) requires Sertoli cells. The synthesis and release of steroid hormones from Leydig cells, and the maturation of Sertoli cells, is closely-regulated by two peptide hormones, luteinizing hormone (LH) and follicle-stimulating hormone (FSH), secreted by the anterior pituitary. LH and FSH circulate in plasma and bind selectively to their specific GPCRs, where they activate adenylyl cyclase, raise intracellular cAMP levels, and activate PKA (**Figure 1**). In the short term, the major effect of LH and FSH on Leydig and Sertoli cells is to stimulate steroid hormone synthesis and release, and spermatogenesis, respectively. However, in the long term, both peptide hormones have essential roles in stimulating the growth and differentiation of their respective target cells.

The biochemical action of PDE11 in testis is essentially identical to that seen in thyroid and adrenal cells, as described above: it hydrolyses cAMP (and, some contexts, cGMP). Therefore, in testis physiology, it has the potential to reverse the actions of LH and FSH. Conversely, loss or inactivation of PDE11 increases cAMP levels and thereby acts in synergy with the hormone-secreting, pro-proliferative actions of LH and FSH. This phenotypic effect of loss-of-function *PDE11A* mutations is observed in patients with Carney Complex and *PRKAR1A* mutations who develop Sertoli cell tumors, as described in more detail below.

Loss-of-function germline *PDE11A* mutations have also been implicated in testicular germ cell tumors [NSGCT; refs (70, 84–88)], which are biologically and clinically very different from Sertoli cell tumors. *PDE11A* mutations in NSGCT are typically single amino-acid mutations that reduce the enzymatic activity and/or protein stability of the PDE11A protein (70, 84, 87). Inactivation of *PDE11A* by increased promoter methylation has also been associated with a familial predisposition to NSGCT (86). *PDE11A* appears to act as a tumor suppressor gene in NSGCT, as NSGCT patients with *PDE11A* mutations are more likely to have a family history of NSGCT and/or to present at a younger age, and/or to present with bilateral tumors (84, 85). Mice with *pde11a* knockouts develop testicular atrophy, which is known to predispose patients to NSGCT (89). Much remains to be learned about how *PDE11A* loss-of-function mutations contribute to the molecular mechanisms of NSGCT pathogenesis (i.e., do *PDE11A* mutations work primarily in Leydig and/or Sertoli cells, and thereby alter the testicular hormone environment, or do they act directly on germ cell precursors, or both)? Further study of these questions should provide additional support for the role of PDE11A as a tumor suppressor and our knowledge of germ cell proliferation and development.

### Mutations of PKA in endocrine tumors and related cancers

#### Carney Complex - PKA mutations in adrenal hyperplasia and tumors

**Figure 2:**
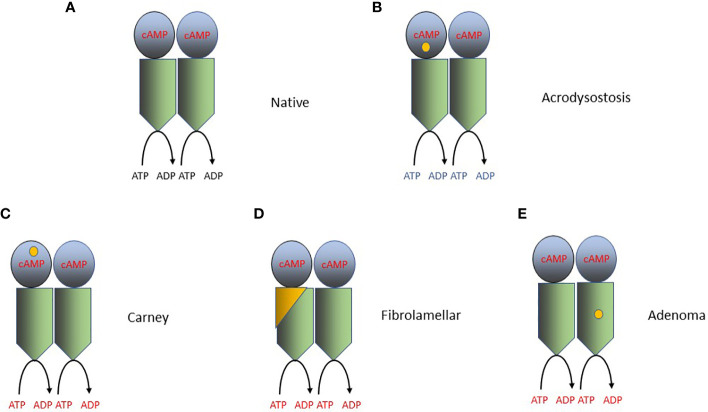
Mutations that activate PKA in cancer and other disorders. PKA catalyzes the phosphorylation of serine and/or threonine amino acids located at specific sites in its protein substrates. ATP is the phosphodonor and therefore the reaction converts ATP to ADP. The PKA holoenzyme is a tetramer of 2 catalytic subunits (C-subunits, Cα or Cβ; blue in the figure), and 2 regulatory subunits (R-subunits, RIα, RIβ RIIα or RIIβ; green in the figure). The RIα subunit is encoded by *PRKARA1A* and the Cα or Cβ subunits are encoded by *PRKACA* and *PRKACB*, respectively. Each R subunit forms a homodimer through the interaction of helices at its amino terminus and also interacts with at least one C subunit. cAMP binds to a specific domain located within each R subunit and produces a conformational change in the PKA holoenzyme, increasing its catalytic (protein kinase) activity. The location of the specific driver mutations in PKA implicated in oncogenesis are shown in each panel by a yellow dot. **(A)** The native (wild-type) PKA holoenzyme. **(B)** Mutations producing acrodysostosis. These mutations are localized to the second cAMP-binding domain of RIα, where they perturb the switch between the active (cAMP-bound) and inactive conformations of PKA. The mutations produce cAMP-resistant PKA holoenzymes and thereby reduce PKA activity. **(C)** Mutations producing Carney Complex. Mutations in one of several amino acids in RIα reduce the stability of RIα or its interaction with Cα/β and therefore increase PKA action. **(D)** Mutations in fibrolamellar hepatocellular carcinoma. The DNAJB1-PRKACA fusion (yellow and green) is capable of binding to RIα and RIIα subunits, creating in each case a functional holoenzyme. DNAJB1-PRKACA holoenzymes are more abundant in cells, producing increased PKA enzymatic activity. **(E)** Mutations in adrenal adenomas. Mutations in the Cα subunit, such as L205R, alters the ability of the Cα subunits to be regulated by the R subunits, leading to constitutive, cAMP-independent signaling. The L205R and W196R mutations also appear to exclude the mutant holoenzymes from their AKAP anchors, allowing them to diffuse indiscriminately throughout the cell, producing phosphorylation of non-physiologic PKA substrates.

Carney Complex is a multi-system disorder that shows both variable penetrance and variable expressivity (i.e., varying clinical manifestations, sometimes called pleiotropy) among affected individuals. The manifestations of Carney Complex include hyperplasia/adenomas of the cortex of the adrenal gland, cardiac and other myxomas, spotty skin pigmentation (lentiginosis), and as well as numerous other abnormalities (51, 90–93). Carney Complex is caused by germline mutations in *PRKAR1A* (which encodes the PKA regulatory RIα subunit) and, rarely, *PRKACA* or *PRKACB* [which encode the PKA catalytic subunits Cα or Cβ, respectively, **Figures 1**, **2**, refs (94–96)]. Germline/mosaic mutations in *PRKACA* or *PRKACB* also cause a complex congenital malformation syndrome, including some unusual tumors, that is phenotypically distinct from Carney complex (97). Given the role of *GNAS* and *PDE11A* mutations in the development of adrenal hyperplasia and adenomas, as described in detail above, and that *GNAS*, *PDE11A* and *PRKAR1A* all encode essential components of cAMP signaling pathways (**Figure 1**), it is not completely surprising that Carney Complex patients develop primary pigmented nodular adrenocortical disease, which may cause Cushing syndrome, and cortisol-secreting adrenal adenomas (91). However, clinically there are differences between patients with Carney Complex and *PDE11A* mutation carriers, as the extra-adrenal features typical of Carney Complex are usually not present in *PDE11A* carriers.

There are also some intriguing genetic interactions between *PRKAR1A* and *PDE11A* in the pathogenesis of adrenal cortical adenomas. A significant proportion of patients with Carney Complex with *PRKAR1A* mutations also have germline loss-of-function mutations in *PDE11A* (98). This interaction is more commonly seen in Carney Complex patients with adrenal hyperplasia, in that patients with adrenal hyperplasia (i.e., primary pigmented nodular adrenocortical disease) were significantly more frequently carriers of *PDE11A* variants than those without adrenal hyperplasia (98). These clinical observations are certainly consistent with these 2 mutations interacting in a common pathway, leading to PKA activation, in these tumors (98, 99). In this context, *PDE11A* can be viewed as a genetic modifier for *PRKAR1A* mutants, a function also seen with several other genes, such as *ARMC5* (100).

#### Somatic PKA mutations in adrenal adenomas

Further evidence for activation of cAMP signaling pathways in adrenal adenoma has come from the study of acquired (somatic) mutations in adrenal adenoma tumor tissue. Exome sequencing of sporadic cortisol-secreting adrenal adenomas has shown a substantial number of them to contain single-amino mutations in *PRKACA*, the most common of which is L205R [ refs (101, 102).; previously known as L206R]. The functional effect of these mutations is to increase PKA activity (the exact mechanism will be described in more detail below). Of the patients with adenomas whose tumors lack mutations in *PRKACA*, a number contain a copy-number gain on chromosome 19 that included *PRKACA*, leading to increased PKA activity (101). Collectively, these observations provide strong evidence for the pivotal role of PKA activation in sporadic cortisol-secreting adrenal adenomas.

#### Carney Complex and PDE11A mutations in tumors of the testis

One of the many manifestations of Carney Complex is the development of large-cell calcifying Sertoli cell tumors [LCCSCT; (90)]. In addition to their *PRKAR1A* mutation, LCCSCT patients with Carney Complex have an increased frequency of germline loss-of-function mutations in *PDE11A* (98). The age of onset tends to be lower, and the incidence and severity of tumors is higher, in patients with mutations in both genes. As is the case for adrenal adenomas, as discussed 2 paragraphs above, these observations provide a clinical correlate of the experimental data for these 2 gene abnormalities working in a common pathway.

#### Carney Complex and pituitary adenomas

Patients with Carney Complex can develop somatotropinomas (46, 90, 92, 103–105) and occasionally other types of pituitary adenomas (106). Consistent with the concept of variable expressivity, up to three-quarters of Carney Complex carriers show abnormal growth hormone response to stimuli such as glucose or TSH-releasing hormone, even without clinical evidence of an adenoma (107). Intriguingly, *PRKAR1A* mutations are not seen in sporadic somatotropinomas (i.e., those not associated with a germline mutation predisposing to adenoma formation), although somatic mutations in *GNAS*, as well as other genes (including *USP8, BRAF,PIK3CA* and *TP53*), are seen commonly in these tumors (46, 108–112).

#### Murine models of Carney Complex and PDE11A loss provide insights into the human diseases

Mice with a range of *Prkar1a* mutations show abnormalities consistent with those seen in humans with Carney Complex. Whole-animal homozygous knockouts of *Prkar1a* are lethal, but tissue-specific *Prkar1a* knockouts in each of pituitary, adrenal cortex, and pancreatic neuroendocrine tissue develop hyperplasia and adenomas in these tissues, respectively (113–115). *Prkar1a* haplo-insufficient mice also develop an increased number of tumors when bred to *Trp53* +/- or *Rb* +/- mice, respectively (116). Mice with a partial inactivation of *Pde11a* develop adrenal subcapsular hyperplasia with predominant fetal-like features in the inner adrenal cortex and have abnormal regulation of cortisol secretion (117). Collectively, these murine models produce additional support for the functional significance of *PRKAR1A* and *PDE11A* mutations in the development of adrenal tumors and that their gene products act in a common pathway.

#### Carney Complex PRKAR1A mutations activate PKA by attenuating the action of the regulatory subunit

The PKA holoenzyme is a tetramer composed of 2 catalytic subunits and 2 regulatory subunits (**Figures 1**, **2**). Each catalytic subunit catalyzes the addition of phosphate (derived from ATP) to serines and/or threonines located in specific sequence motifs present in the substrates of the enzyme. The PKA catalytic regions are functionally and structurally homologous to those present in other protein kinases, especially closely-related serine-threonine kinases, such as AKT and PKC. Each PKA regulatory subunit binds cAMP and interacts with the other regulatory subunit and with at least one catalytic subunit (118). The subcellular localization and many other aspects of the regulation of PKA are in turn regulated by A-kinase anchoring proteins (AKAPs) that bind to the regulatory subunits (22). In the resting holoenzyme, the regulatory subunits bind to, and inhibit, the catalytic subunits. Binding of cAMP to the regulatory subunits changes the conformation of all four subunits, thereby activating their catalytic activity (20). cAMP regulation of PKA activity is therefore a very dynamic process, regulated closely in both space and time.

The vast majority of PKA mutations in Carney Complex are in the regulatory RIα subunit (encoded by *PRKAR1A*). However, rare Carney Complex mutations are found in the catalytic Cα or Cβ subunits (encoded by *PRKACA* or *PRKACB*, respectively). The *PRKAR1A* mutations in Carney Complex activate PKA by attenuating the abundance or action of the regulatory subunit (94, 95, 119). The majority of *PRKAR1A* mutations reduce the abundance of *PRKAR1A* mRNA by triggering nonsense-meditated mRNA decay, leading to haploinsufficiency (120). Other *PRKAR1A* mutations encode amino acid substitutions, in-frame alterations, or frame-shifts (120). Although the functional significance of many of the amino acid substitutions has yet to be studied, it appears that many of them reduce the interaction of the RIα subunit with the Cα and/or Cβ subunit, leading to aberrant over-activity of the Cα and/or Cβ subunit.

Additional insights into the relationship of the RIα and Cα and/or Cβ subunits in Carney Complex has come from study of a very different inherited disease, acrodysostosis. Acrodysostosis is an inherited disorder of bone formation and cognition produced by mutations in either *PRKAR1A* or the cAMP-specific phosphodiesterase *PDE4D* [see ref (9)., especially the Supplemental text file, for a review]. Patients with acrodysostosis do not appear to have an increased susceptibility to cancer. The precise mutations in *PRKAR1A* in acrodysostosis are different from those seen in Carney Complex. Acrodysostosis *PRKAR1A* mutations are located in the second cAMP-binding domain of RIα, where they perturb the switch between the active (cAMP-bound) and inactive conformations of PKA. Carney Complex *PRKAR1A* mutations reduce the stability of RIα and therefore increase PKA action, while acrodysostosis *PRKAR1A* mutations result in cAMP-resistant PKA holoenzymes and thereby reduce PKA activity (119, 121). Study of acrodysostosis mutations has provided new insight into how the “dynamically controlled crosstalk between the helical domains of the two [cAMP-binding] domains is necessary for the functional regulation of PKA activity” (119).

### Mutations of the PKA catalytic subunit in fibrolamellar hepatocellular carcinoma

Fibrolamellar hepatocellular carcinoma (HCC) is an uncommon liver cancer, with distinctive pathological and clinical features, that affects primarily adolescents and young adults. In contrast, typical hepatocellular carcinoma arises in older adults in the context of prior cirrhosis and/or viral infection (typically, hepatitis B or C). The tumor cells of virtually all patients with fibrolamellar HCC contain an approximately 400 kilobase deletion that creates an in-frame gene fusion between *PRKACA* and *DNAJB1* (122–125). *DNAJB1* encodes heat-shock protein 40, a molecular chaperone, and the DNAJB1-PRKACA fusion in fibrolamellar HCC is encoded by exon 1 of *DNAJB1* and exons 2-10 of *PRKACA* (122). The DNAJB1-PRKACA fusion (and in at least one case, an ATP1B1-PRKACA fusion) has also been detected in occasional cases of pancreaticobiliary neoplasms, which are histologically and clinically different from fibrolamellar HCC (126). Intriguingly, *PRKAR1A*-mutated Carney Complex patients can also develop fibrolamellar HCC, but their fibrolamellar tumors show loss of PRKAR1A expression and no detectable mutations in *PRKACA* (127). Studies of mice genetically-engineered to express a *Dnajb1-Prkaca* fusion showed that the fusion clearly has a strong oncogenic effect in liver tissue (128, 129).

The DNAJB1-PRKACA fusion mRNA is expressed at significantly higher levels than un-mutated PRKACA mRNA in fibrolamellar HCC, producing overexpression of the fusion protein (124). Compared to normal liver, the expression of RIα protein is increased approximately 2-fold, with no change in RIIα and a decrease in RIIβ protein levels (124, 130). The DNAJB1-PRKACA fusion is capable of binding to RIα and RIIα subunits, creating in each case a functional holoenzyme. Holoenzymes containing the DNAJB1-PRKACA fusion protein have significantly increased cAMP-induced activity, compared to those containing an un-mutated Cα subunit protein. Since both holoenzymes have similar K_m_ for substrate, the increased enzymatic activity appears to reflect the increased abundance of holoenzymes containing the DNAJB1-PRKACA fusion (124).

Structural and enzymatic studies of the DNAJB1-PRKACA fusion protein have yielded important insights into its oncogenic effect in fibrolamellar HCC and have also provided new insights into the regulation of the PKA holoenzyme by its regulatory subunits (**Figure 2**). These studies have shown that the DNAJB1-PRKACA fusion protein is capable of interacting with RIα, producing a holoenzyme that is still capable of being regulated by cAMP (130). One study (131) showed that the DNAJB1 portion of the fusion (called the J-domain) can stabilize the second cAMP-binding domain of the RIIβ subunit, increasing its ability to be activated by cAMP; other studies have failed to confirm this finding (132, 133). Other mechanisms for the effect of DNAJB1-PRKACA fusion protein have also been proposed (134). In contrast, study of the functional effects of the somatic *PRKACA* mutations, especially L205R, in adrenal adenomas shows that the L205R mutation alters the ability of the Cα subunits to be regulated by the regulatory subunits, leading to constitutive, cAMP-independent signaling (101, 102, 130, 135). The L205R and W196R mutations also appear to exclude the mutant holoenzymes from their AKAP anchors, allowing them to diffuse indiscriminately throughout the cell, producing phosphorylation of non-physiologic PKA substrates (135, 136).

The increased cAMP stimulation of PKA in fibrolamellar carcinoma may have several important cellular consequences. The DNAJB1-PRKACA fusion protein appears recruit cellular proteins that do not normally interact with PKA holoenzymes, such as heat-shock protein 70 (hsp70), which in turn allows it to interact with a RAF-ERK-MAPK signaling module (137). This fusion-specific interaction would therefore allow ERK/MAPK signaling in fibrolamellar carcinoma cells to be driven by hormones, such as neurotensin, that elevate cAMP levels and which are elevated in the fibrolamellar HCC microenvironment (138). It also has potential therapeutic consequences, as agents that would lower cAMP levels in fibrolamellar HCC cells would have anti-oncogenic effects.

### Downstream cAMP effectors - beyond PKA

Key to understanding the cellular and organismal effects of cAMP signaling are the mechanisms by which cAMP elevation produces its physiological effects. There is abundant evidence for the essential role of PKA in downstream cAMP signaling in oncogenesis, as described in detail above. However, cAMP has at least three other effectors, EPAC, cAMP-gated ion channels, and popeye proteins (**Figure 1**). EPAC proteins are essential to a number of functions, particularly in the CNS (11, 12); however, EPAC mutations have yet to be identified as drivers in human cancer (139). Cyclic-nucleotide-gated ion channels have essential functions in the CNS, cardiovascular and other systems (13–15), but channel gene mutations have yet to be identified as drivers in human cancer. Popeye proteins appear to have essential functions in skeletal and cardiac muscle and other tissues (17, 19); they have been postulated to have a role in human cancer (16, 18), although popeye mutations have yet to be identified as drivers in human cancer.

### What are the oncogenic targets of PKA action?

PKA has numerous downstream phosphorylation targets, which play an essential role on the regulation of myriad cellular and organismal functions, ranging from metabolism (glycogen), to the CNS (synaptic function and learning and memory). Intensive investigation has provided essential insights into the physiological substrates of PKA in many of these systems, including the CNS [see refs (140–142). for reviews]. The continued application of dedicated phosphoproteomic approaches is highly likely to identify additional candidates (143). Such efforts are just beginning to pay off in cancer generally, and for the cAMP-pathway cancers in particular (136, 137, 144). Abnormal PKA action in cancer has the potential to produce two broad types of phosphorylation abnormalities: (1) increased phosphorylation of normally-physiologic substrates and (2) phosphorylation of new (hence, non-physiologic/pathologic) substrates. Given the complexity of these effects, it is reasonable to focus, at least initially, on phosphoproteins that are encoded by driver mutations in cancer. There are two excellent candidates: CREB family members and BRAF.

### Mutations in CREBs and related proteins in a distinct set of human cancers

cAMP-response elements (CREs) are regions of DNA sequence, typically located in the promoters of eukaryotic genes, that allow gene expression to be modulated by cAMP. CREs act as binding sites for the cAMP-response element binding (CREB) protein, and the closely-related proteins ATF1 and CREM. Binding of CREB family members recruits coactivator proteins, such as CREB-binding protein (CBP or CREBBP) and p300. CBP and p300 have intrinsic histone acetyltransferase activities and serve to recruit additional components of the transcriptional machinery, including RNA polymerase II, to promoters and thereby regulate gene expression. CREB, ATF1 and CREM are important substrates for several protein kinases, including PKA and ERK1/2. These kinases phosphorylate CREB-family transcription factors at a common, single site (S133 in CREB). Phosphorylation of CFEB-family members promotes their transfer to the nucleus, DNA binding, and transcriptional activation (145, 146). CREB family members have numerous essential physiological roles, including the regulation of vital CNS functions related to learning and memory.

Activating mutations in CREB family members have been shown to be driver mutations in two pathologically-distinct sets of human cancers. One group of these cancers is characterized by intra-abdominal epithelial or soft-tissue tumors, where the driver mutation is a fusion between a CREB family member and EWSR1 or FUS [i.e., EWSR1-CREM, FUS-CREM or EWRS1-ATF1 fusions, also called FET-CREB fusions (147–149)]. A second set of these cancers is clinically distinct, arising in the CNS, with similar fusions but a different set of accompanying driver mutations (150, 151). In both groups of these cancers, the CREB-family member is activated by being incorporated in the protein encoded by the fusion and by being placed under the control of the EWSR1 or FUS promoter, both of which are transcriptionally active in the cells of origin of these tumors. The identification of the FET-CREB fusions as drivers in these cancers provides important validation of the role of CREB family members in oncogenesis; however, many questions remain, including the question of whether phosphorylation of the CREB family member incorporated in the fusion is required for the oncogenic activity of the fusion, and, if so, whether that phosphorylation is mediated by PKA, as opposed to ERK1/2, or possibly other kinases. The specific set of genes that is regulated transcriptionally by CREB phosphorylation in cAMP-specific cancers has also yet to be explored fully (152, 153). Broadly speaking, does aberrant CREB action in cancer produce increased levels of transcription of its normally-physiologic “target” genes OR does it increase transcription of “off-target” (i.e., non-physiologic) genes?

### BRAF: driver mutations in a downstream PKA target

Mutations in the RAF family of serine-threonine protein kinases, especially in *BRAF*, are commonly-observed drivers in a diverse range of human cancers (154). The regulation of RAF is a highly-complex process, mediated, at least in part, by dimerization of several RAF family members, including the scaffold protein KSR. The major downstream target of RAF kinases is a MEK-ERK1/2 signaling module, activation of which has numerous oncogenic effects. Members of the RAS family of monomeric G-proteins regulate RAF proteins and are in turn regulated by a membrane-associated signaling complexes that can includes several growth-factor receptor tyrosine kinases. RAS mutations, notably those in *KRAS* and *NRAS*, are among the most commonly-observed drivers in human cancer and are a major focus of drug-discovery efforts (155). Activation of cAMP signaling antagonizes RAS-mediated RAF activation (156–158), leading to reduced activity of the MEK-ERK1/2 cascade. PKA phosphorylates RAF at several serines, most notably S259, producing inhibition of RAF kinase activity (159–162). However, PKA action, mediated by AKAP-Lbc, may enhance ERK1/2 signaling by phosphorylating KSR at S838 (163). Finally, PDE8A is capable of interacting with RAF at high affinity; this interaction lowers local levels of cAMP, inactivates PKA, and thereby activates ERK1/2 (164, 165). Given the complexity and multiple modes of regulation of the RAS-RAF/MEK/ERK1/2 signaling pathway, the physiological consequences of PKA action on this pathway remain uncertain. Discovery of additional driver mutations in the members of the complex would provide compelling and highly-needed confirmation of the roles, if any, of PKA in its regulation.

### Non-coding RNAs and cAMP-specific cancers

Long non-coding RNAs (lncRNAs), defined as RNAs greater than 200 nucleotides in length that do not encode proteins, and microRNAs (miRNAs, miRs) are essential regulators of many components of cAMP signaling. For example, miR-34a directly targets *GNAI2*, which encodes an inhibitory G protein α subunit, as well as other potential regulators of cAMP signaling (166–168). Significant changes in the expression (i.e., tumor v normal) of several different miRNAs have been observed in somatotropinomas (166, 168–173). Specific lncRNA expression signatures have been identified in adrenal adenomas and carcinomas and may have clinical value in distinguishing these 2 neoplasms in tissue specimens (174, 175). Differential expression (tumor v normal) of miRNAs and lncRNAs has also been observed in fibrolamellar hepatocellular carcinoma, although it currently unclear whether these RNAs regulate cAMP pathway components, as opposed to other drivers, in these cancers (152, 176). To date, however, driver mutations in miRNAs and/or lncRNAs have yet to be identified in cAMP-signaling pathway cancers (166–173). Further whole-genome sequencing of diverse tumor types, followed by functional analysis of the mutations identified in these screens, are necessary before we can conclude that miRNA or lncRNA mutations are truly capable of acting as drivers in these cancers.

## New insights and areas for future research

The expanding universe of cAMP-pathway cancers has provided new insights into the concept of driver mutations in these and other cancers. Originally, the dichotomy between driver and passenger mutations in cancer was made on the basis of genomic/bioinformatics approaches, as described above. However, the ever-increasing structural, enzymatic and pharmacologic data on cAMP pathway signaling components now compels investigators in this field to determine how driver mutations affect the structural, enzymatic and pharmacological properties of each of these signaling proteins. As first demonstrated by the pioneering studies on RAS oncoproteins and protein-tyrosine kinases in cancer, the ongoing structural and enzymatic studies on cAMP pathway components need to confirm that these mutations are of sufficient functional importance to be indeed designated as drivers, as opposed to passengers. Recent progress in this field, as described above, has been very gratifying and has provided a wealth of well-verified driver mutations worthy of intensive investigation.

The concept of driver mutations also helps to explain one of the most interesting paradoxes in this field: how do various mutations, all activating a common signaling pathway, produce such a diversity of cancer phenotypes? In some cases, the precise pattern of mutations in a given protein in each of several diverse cancers is quite different [e.g., the different PKA mutations in each of Carney Complex, adrenal adenomas and acrodysostosis (**Figure 2**)], consistent with each mutational pattern having the intrinsic ability to produce a distinct cancer phenotype. However, for other drivers (e.g., *PDE11A*), there is no obvious difference in the mutational pattern of the driver in various clinically and biologically diverse cancers. In these latter cases, the context of the driver mutation is critical. Exome sequencing has shown the presence of additional driver mutations in *PDE11A*-associated cancers and, more importantly, that these additional driver mutations differ substantially from one cancer to another (e.g., the pattern of driver mutations [and other genomic events, such as methylation] observed in adrenal adenomas is extremely different from those seen in testicular cancer). Functionally, these differences in context help to explain the clinical and biological differences in cancers that contain the same driver mutation (e.g., the diverse cancers with mutations in *PDE11A* or with those with mutations in *CREB/ATF/CREM*). Further genomic analysis of these cancers (i.e., deep sequencing, analysis of epigenetic changes, and gene expression patterns) is highly likely to produce additional examples of the importance of context (28).

Related, but mechanistically separate, to the concept of mutational “context” is the importance of differential expression of the various pathway components in the tumor cell of origin. Tumor stem cells differ from their corresponding normal tissue/organ stem cells in that they contain a discrete pattern of driver mutations, but are similar to their normal counterparts in that they retain similar/overlapping gene expression patterns. These similarities in gene expression are reflected in the phenotypic similarities between many cAMP-signaling cancers and their corresponding tissue/organ or origin; for example, adrenal cortical adenomas are similar to normal adrenal cortical tissue in their ability to produce steroid hormones. The most likely explanation for this overlap is that tumor stem cells retain many of the gene expression patterns of the corresponding normal stem cells, presumably by the retention of transcription factors, methylation patterns, and other regulators of gene expression found in the normal stem cells. Given that cAMP-signaling cancers arise in many distinct, differing tissue types, this ability of tumor stem cells to “remember” their original stem cell of origin appears to largely account for their incredible diversity of cancer phenotypes, even though their mutations affect a common signaling pathway.

A final explanation for the diversity of the cAMP-signaling cancers is the distinct patterns of mutations in each of the pathway components that is seen in each different tumor type. This is best appreciated for the different patterns of mutations in the PKA holoenzyme seen in various cancers (**Figure 2**). Each of these mutation patterns has a distinct effect on the biochemical regulation of the pathway components. As we have seen most convincingly for PKA, these varying regulatory effects are likely to have a profound influence on the phenotype of each individual cancer. It is likely that further studies of the biochemical effects of the various mutations, especially on proteins, such as PDE11A, that are mutated in a wide range of different cAMP-pathway cancers, will provide additional insights into the basic mechanisms of regulation of these pathway components.

The identification of functionally-significant driver mutations in cancer provides a major impetus towards the development of pharmacologic agents that target these mutations. In theory, the identification of an increasingly well-characterized mutational pattern (both passengers and drivers) in human cancer generally would, at first glance, appear to provide a “target rich” environment for therapeutic development. However, despite advances in therapeutics generally, many driver mutations in cancer appear to be essentially “undruggable”. To date, there are no clinically-available drugs that target cAMP-signaling components that have been proven to successfully treat cAMP-signaling cancers. However, ongoing drug discovery efforts have the potential to change this. Given the success of small-molecule inhibitors of tyrosine protein kinases in cancer therapeutics (which target the ATP-binding site of these enzymes), the development of clinically-useful PKA inhibitors that target their ATP-binding site would seem to be a logical first step (177). The recent development of activators, especially allosteric activators, of PDE4s (178) has provided an impetus for the development of activators of other PDE classes. Activators of PDE8 and PDE11 would have potential value in the treatment of tumors with hemizygous loss or inactivation of *PDE8B* of *PDE11A*, respectively, and potentially in other cAMP-pathway cancers characterized by elevated levels of cAMP. Specificity of action remains important; for example, the recent observation that PDE4D inhibition exerts anti-oncogenic action in liver cancer would be unlikely to apply to fibrolamellar hepatocellular carcinoma (179). The success in drug discovery targeting GPCRs generally is grounds for optimism that effective drugs targeting the TSH receptor or GRP101 may be developed in the future. Finally, evolving drug discovery technology that targets genes or RNAs (e.g., CRSPR, siRNA or antisense), rather than proteins, offers new approaches to targeting these otherwise undruggable targets.

## Author contributions

The author confirms being the sole contributor of this work and has approved it for publication.

## Funding

This work was supported by BZI Pharma LLC.

## Conflict of interest

The author is an employee of, and shareholder in, BZI Pharma LLC, which had no role in the design or execution of the study, in the writing of the manuscript, or in the decision to submit it for publication. The author has no other conflicts of interest.

## Publisher’s note

All claims expressed in this article are solely those of the authors and do not necessarily represent those of their affiliated organizations, or those of the publisher, the editors and the reviewers. Any product that may be evaluated in this article, or claim that may be made by its manufacturer, is not guaranteed or endorsed by the publisher.
